# The Young, the Weak and the Sick: Evidence of Natural Selection by Predation

**DOI:** 10.1371/journal.pone.0009774

**Published:** 2010-03-19

**Authors:** Meritxell Genovart, Nieves Negre, Giacomo Tavecchia, Ana Bistuer, Luís Parpal, Daniel Oro

**Affiliations:** 1 Population Ecology Group, Department of Biodiversity and Conservation, IMEDEA (CSIC-UIB), Esporles, Spain; 2 Fundació Natura Parc, Santa Eugènia, Spain; 3 Servei de Gestió de Residus, Consell de Mallorca, Palma de Mallorca, Spain; 4 Consorci per a la Recuperació de la Fauna de les Illes Balears (COFIB), Santa Eugènia, Spain; University of Oxford, United Kingdom

## Abstract

It is assumed that predators mainly prey on substandard individuals, but even though some studies partially support this idea, evidence with large sample sizes, exhaustive analysis of prey and robust analysis is lacking. We gathered data from a culling program of yellow-legged gulls killed by two methods: by the use of raptors or by shooting at random. We compared both data sets to assess whether birds of prey killed randomly or by relying on specific individual features of the prey. We carried out a meticulous post-mortem examination of individuals, and analysing multiple prey characteristics simultaneously we show that raptors did not hunt randomly, but rather preferentially predate on juveniles, sick gulls, and individuals with poor muscle condition. Strikingly, gulls with an unusually good muscle condition were also predated more than expected, supporting the mass-dependent predation risk theory. This article provides a reliable example of how natural selection may operate in the wild and proves that predators mainly prey on substandard individuals.

## Introduction

Predation is an important selective force in evolution [1]–[7] and is generally assumed to select against substandard individuals, i.e. the young, senescent, sick, or individuals in poor physical condition [8]–[9]. Although some studies support this hypothesis and have contributed substantially to the understanding of selection by predation [10]–[20], (but see [18]), most of them are based on opportunistic observations or rely on some specific traits of the prey [15], [18]–[20] or parasite load [12], [13]. Kenward [10] and Temple [11] investigated morphological traits of individuals together with their healthy state, however, sample sizes were rather small (less than 30 prey) and the effects of different traits were not analysed simultaneously, so the contribution of each trait on differential predation it is difficult to evaluate. Additionally, all evidence typically comes from the typical predator-prey system, where traits may have coevolved in parallel, and thus predation upon substandard individuals could be an opportunistic foraging strategy rather than a response to substandard features of the prey. To fully understand the role of predation as a selective force, it is also necessary to collect evidence of predation outside the typical predator-prey system, gather information on large sample sizes, and investigate multiple traits of prey simultaneously.

Populations of large gulls have increased substantially over the last century and some species are currently perceived as a pest by wildlife managers [21]–[24] but see [25]. As a consequence, many conservation agencies have set up culling programs to control gull populations, which typically consist of the systematic removal of large numbers of eggs, chicks or breeding adults. Even if the efficacy of these culling programs is still under debate [25]–[28], many conservation agencies still control gulls by culling. The Local Government of the Balearic Islands (Spain) began a gull culling programme on a refuse tip in the island of Mallorca as a part of the population control of yellow-legged gulls -*Larus michahellis*- in the Balearic archipelago. From 2003 to 2007 birds were culled by two methods: by shooting or by the use of trained birds of prey (peregrine falcon -*Falco peregrinus*-, saker falcon -*F. cherrug*- and Harris's hawk -*Parabuteo unicinctus*-). We gathered data from this culling program and examined killed birds to determine 1) the sex and age of the individual, 2) individual body condition, assessed from muscle condition, and 3) any sign of parasitism (internal and external), infection, malformation or chronic disease (e.g. aspergillosis). We used these data to investigate multiple prey traits simultaneously and to assess whether birds of prey killed randomly or by relying on specific individual prey features.

## Results

**Figure 1:**
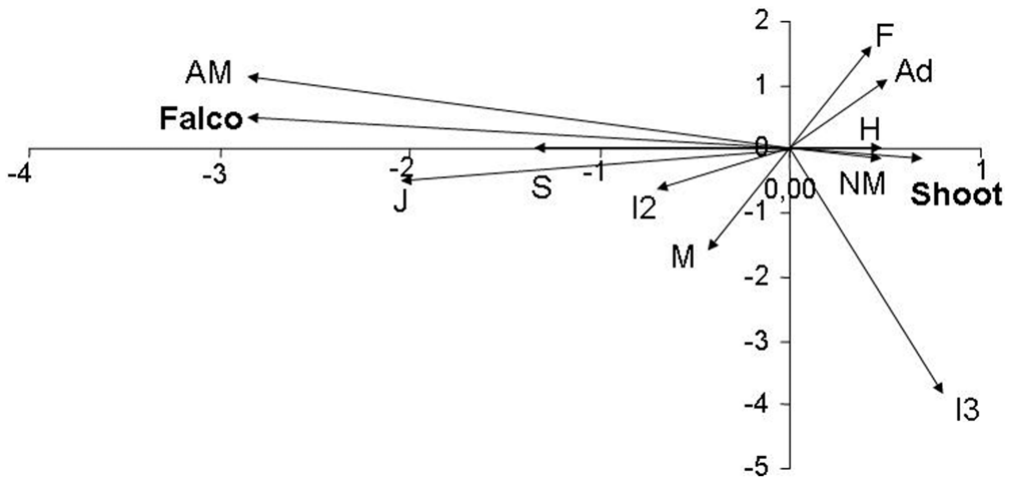
Multiple Correspondence Analysis between individuals shot and those killed by raptors. Map of the two main factorial axes from a Multiple Correspondence Analysis between individuals shot (noted by Shoot) and those killed by birds of prey (noted by Falco) depending on individual classification (F: Females; M: Males; J: Juveniles; I2: 2 years old; I3: 3 years old; A: Adults; H: Healthy individuals; S: Sick; AM: Abnormal muscle condition (low and high); NM: normal muscle condition).

**Figure 2:**
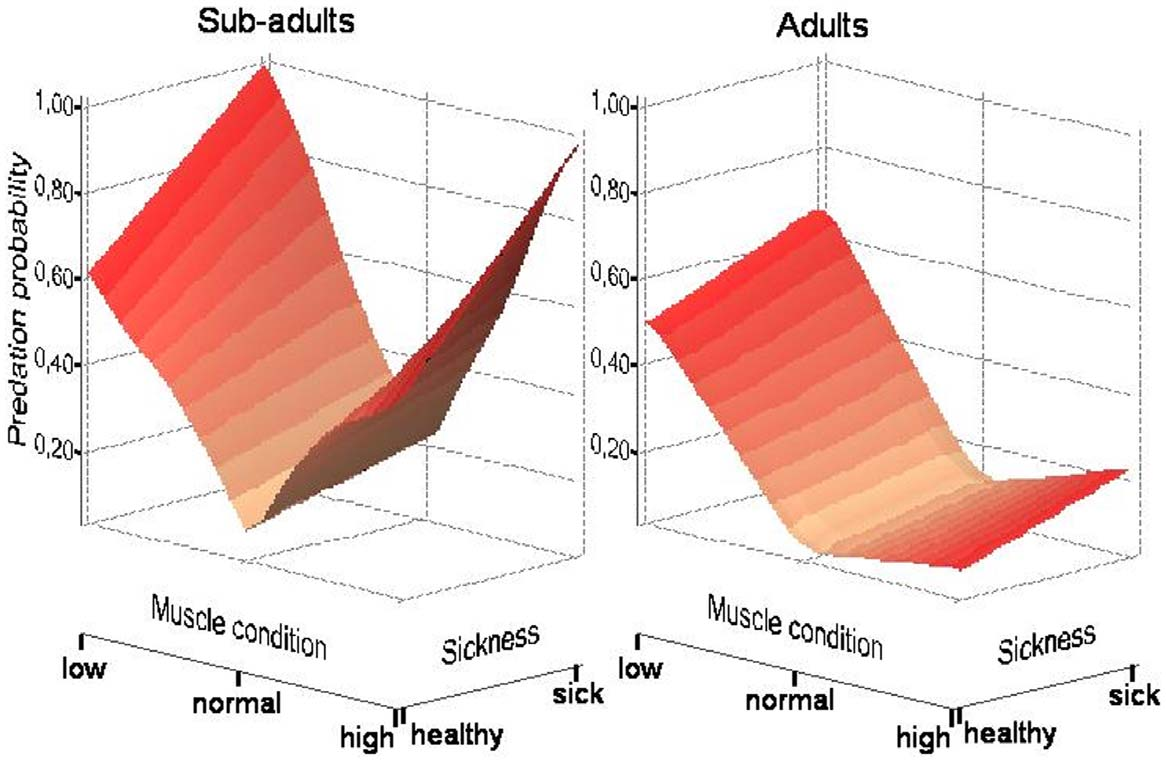
Determinants of probability of being predated by the raptors. All juveniles and immature classes were grouped in a single, sub-adult age class and compared with adult gulls. Smoothing regression surfaces are represented using a Lowess method by iteration of weighted least squares on the selected variables. Highest probability of being killed by predators occurred on sub-adult gulls with severe sicknesses and abnormal muscle condition.

We examined 506 gulls that had been shot and 122 gulls removed by raptors. The age structure in the shooting sample was similar to that observed at the dump over five available censuses (Breslow-Day homogeneity test of odds ratio, 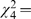
3.311, P = 0.507; Mantel-Haenszel, odds-ratio -log transformed- 95% Confidence Intervals: [−0.066; 0.102], see Table 1). Post-mortem examination of gulls (Table 2) showed a low prevalence of external parasites; however, half of the individuals examined had internal parasites, mainly cestodes. About 7% of the gulls showed infection by *Salmonella* and 4% by *Aspergillus*. A few individuals also showed some kind of congenital malformation (bill deformity) and others had alteration of internal organs (Table 2), but prevalence of most veterinarian findings were low. Individuals that had been shot showed different characteristics than those killed by raptors (Table 3). The Multiple Component Analysis (MCA) scores plot also showed differences between groups, with more healthy adults and a higher average muscle condition within the group of individuals shot, and more juveniles, gulls in poor condition or showing some signs of illness in the group of individuals killed by birds of prey (Fig. 1). To test for the significance of these differences we used logistic regressions with predation by raptors as the response variable, being “killed by raptor” = 1 and “killed by shooting” = 0. In this way we retained shooting as the intercept of the regression to check for differential predation. The overdispersion value of the saturated model was 1.09, indicating a good fit of the data. The best ranked model (based on AIC values, Table S1) included a negative effect of age and a positive effect of both sickness and poor muscle condition on the probability of being predated by birds of prey (Table 4); this model did not include a gender effect. Strikingly, individuals in unusually good condition were also predated more frequently than expected by chance (Table 4). A model including an effect of sex explained the data equally well and was statistically equivalent to the previous model (Table S1) but the effect was not significant (*z* = −1.293, P = 0.196). Note that all models with lower AIC values unequivocally showed that age, muscle condition and sickness were clues for differential predation by birds of prey (see also Fig. 2). When these three variables were tested separately, results showed that muscle condition was the main factor affecting predation, this variable alone explaining 71% of the total variance.

**Table 1:** Frequencies of gulls of each age-class (sub-adults and adults) counted during the censuses and compared with those of gulls shot at the dump.

			method		
Census no.			census	shooting	Total
1	age	sub-adults	224	821	1045
			31.1%	32.5%	
		adults	496	1705	2201
			68.9%	67.5%	
	Total		720	2526	3246
2	age	sub-adults	224	267	491
			31.1%	28.9%	
		adults	496	657	1153
			68.9%	71.1%	
	Total		720	924	1644
3	age	sub-adults	224	460	684
			31.1%	30.3%	
		adults	496	1058	1554
			68.9%	69.7%	
	Total		720	1518	2238
4	age	sub-adults	224	1118	1342
			31.1%	29.0%	
		adults	496	2737	3233
			68.9%	71.0%	
	Total		720	3855	4575
5	age	sub-adults	224	481	705
			31.1%	32.8%	
		Adults	496	987	1483
			68.9%	67.2%	
	Total		720	1468	2188

Frequencies and percentages of each age-class were shown separately for each census period.

**Table 2:** Results of the exhaustive post-mortem examination of culled gulls (N = 506 and 122 for shooting and caught by raptors respectively).

Veterinarian findings	Prevalence	
	Shooting	Raptors
External parasites		
Lice	0.83	8.20
Ticks	0.14	0
Mites	0.56	0
Internal parasites		
Nematode	4.58	8.20
Cestode	47.08	43.44
Infections		
Salmonella	7.36	1.64
Aspergillosis	0.28	22.95
Retromandibular abscess	0	0.82
White spots on liver	0.14	0
White spots on intestinal wall	0	0.82
Lesions from mites	0.14	0
Internal organ findings		
Atrophies	0	0.82
Lung granulomes	0	0.82
Pericarditis	0.14	0
Pancreas congestion	0.14	0
Hepatomegaly	0.56	0.82
Splenomegaly	7.22	5.74
Peritonitis	0.14	0
Airsacculitis	0.42	0.82
Mechanical dysfunctions		
Traumatism	1.53	33.61
Bill deformity	0.14	0.82
Fishing hooks	0	0.82
Arthritis	0.14	0.82

**Table 3:** Individual traits of gulls removed by shooting or by the use of birds of prey.

Category	Level	Type of disposal		
		Shooting (N)	Raptors (N)	Total N
**Age**	Juveniles	9.68% (49)	36.88% (45)	94
	2-year-olds	11.07% (56)	18.85% (23)	79
	3-year-olds	13.83%(70)	4.09% (5)	75
	Adults	65.41% (331)	40.16% (49)	380
**Sex**	Males	46.8% (237)	60.7% (74)	311
	Females	53.2% (269)	39.3% (48)	317
**Muscle condition**	Normal	89.9% (455)	37.7% (46)	501
	Low	5.1% (26)	49.2% (60)	86
	High	4.9% (25)	13.1% (16)	41
**Health**	Good	78.7% (398)	50.0% (61)	459
	Mild sickness	6.9% (35)	19.7% (24)	59
	Severe sickness	14.4% (73)	30.3% (37)	110
		506	122	628

Removed birds were examined to determine the sex and age of the individual, the individual nutritional state, assessed from fat layers and muscle condition, and any sign of infection, malformation or disease. We identified four age classes by plumage features: juveniles (from 0 to 1 year old), 2 years old (from 1 to 2 years old), 3 years old (from 2 to 3 years old), and adults (>3 years old). We determined three levels of body condition: normal, low and high, depending on the layers of muscle mass). Veterinarians determined if the illness detected was either severe or mild.

**Table 4:** Estimates of the factors affecting predation from the best ranked model, which included age as a continuous covariate, muscle condition as a factor with three levels (normal, low and high), and sickness, as a factor with two levels (healthy and sick).

		Estimate	Std. Error	z value	Pr(>|z|)
Intercept		−0.95	0.35	−2.76	0.0058
Age		−0.59	0.11	−5.63	<0.0001
Muscle condition	Low	3.22	0.32	10.17	<0.0001
	High	1.79	0.40	4.46	<0.0001
Sickness		1.20	0.27	4.42	<0.0001

## Discussion

Natural selection of certain prey traits (e.g. morphological traits) has repeatedly been shown to be driven by predation [10]–[20]. Our paper could not address such particular issue, but on the other hand, the exhaustive analysis of a large number of prey, combined with the simultaneous analysis of a variety of traits, give us some general insights into how predation may operate in the wild. Here we show that predators did not kill individuals at random, but rather selected their prey on the basis of several, not always related traits. Our results indicated that age, muscle condition and sickness influence the probability of being predated, with juveniles, sick gulls, and individuals with poor muscle condition being killed preferentially; thus strongly supporting the hypothesis that predators prey primarily on substandard individuals [8], [9].

Natural selection acts on many characters simultaneously [29] and accordingly, our results dealing with natural selection by predation would suggest that two or more traits may affect fitness in an interactive way (i.e. correlational selection) [7], [29], [30]. Nevertheless, even if our sample sizes were relatively large, they still lack sufficient power to simultaneously test interactive effects between many characters.

Our study also shows that not only individuals with severe diseases but also those with mild diseases are predated preferentially, indicating that subtle changes in behaviour or condition may have been sufficient to increase susceptibility to predation. This was also found by Miller et al (2000) who showed that prion infection in deer increased the rate of predation of deer by mountain lions (*Puma concolor*) nearly fourfold, even if few of the deer killed were recorded as “noticeably ill” by field observers prior to their deaths [31].

Our results clearly support the mass-dependent predation risk (MDPR) theory, which predicts that birds should keep their mass as low as possible to reduce their likelihood of being killed by predators [32], [33]. To date, empirical evidence for this theory comes only from small passerines with body masses between 10–150gr [34,35 and references therein] and recently from one mammal [36]. Additionally, results showed preferential predation on those individuals with poor muscle condition, suggesting that stabilizing selection [37] could be operating on traits linked to body mass.

This article provides a reliable, robust example of how natural selection by predation operates in the wild and strongly supports the paradigm that predators kill substandard individuals. Since gulls are an occasional prey of falcons and hawks, results probably indicate the ability of predators to detect substandard individuals in the wild rather than showing an optimal foraging strategy or possible coevolution within a natural predator-prey system.

## Materials and Methods

From 2003 to 2007, we carried out 5 gull censuses at the landfill and estimated the proportion of birds in each age class. We identified four age classes by plumage features: juveniles (from 0 to 1 year old), 2-year-olds (from 1 to 2 years old), 3-year- olds (from 2 to 3 years old), and adults (>3 years old); these data were used to assess whether shooting was performed randomly regarding to age, by means of a goodness-of-fit test. We cannot exclude that shooting was biased in relation to veterinarian findings (health state) and muscular condition; however if a bias existed it would rend our comparison more conservative as individuals that were ill or in poorer condition should be shot preferentially [38].

Post-mortem examination of culled individuals was carried out at the Fundació Natura Parc-COFIB wildlife recovery centre in Mallorca. Within each age-by-sex class, we sorted individuals as healthy, mildly sick or severely sick depending on the veterinarian diagnosis on infections, mechanical or internal dysfunctions; individuals were also classified according to three levels of body condition depending on the pectoral musculature mass: normal, low or high (extremely large muscle mass). In subsequent analyses, sickness was treated in two ways, one by separating mild and severe sickness and a second one by grouping all sick individuals into a single group.

Gulls breed in springtime and differences may exist in the number of individuals of different sex or age visiting the dump throughout the year, so we defined two periods: one encompassing the breeding season, from March to July, and a second one including the non-breeding period, from August to February. The number of gulls captured by falcons and those killed by shooting were not equally distributed throughout the year, with comparatively more gulls shot during the breeding period. As a consequence, we randomly balanced the sample size between both periods to assure the comparability of the two data sets.

Individuals were shot at random (see above), with the same probability for all birds at the dump to be shot and shooting did not occur in a spatially segregated manner. Hence, comparison between the birds shot and those killed by falcons should reveal any predator preferences. We first used a MCA analysis including age, muscle condition, and sickness to visualize patterns of differentiation among gulls depending on the type of capture and the correlation among traits. We then used logistic regressions to test for differences between the two groups. We assessed the goodness-of-fit of the saturated model by estimating the overdispersion parameter (a value close to 1 indicated a good fit of the data). Model selection was based on Akaike's Information Criterion (AIC); the model with the lowest AIC value was considered as the best compromise between model deviance and model parameters [39]. We also calculated the AIC weight as a measure of relative plausibility of each model. To avoid model over-parameterization, only additive models were considered and individual age was treated as a continuous covariate. All statistical analyses were done using the software R (www.r-project.org) and SPSS (version 16.0).

### Ethics Statement

Animals were killed as a part of a culling program that the Local Government began for conservation issues as well as public health concerns. All animal work has been conducted according to relevant national and international guidelines and permits were provided by Conselleria de Medi Ambient (Govern Balear). Authors were not responsible nor executed the culling programme.

## Supporting Information

Table S1 Model selection of individual features of gulls predated by birds of prey compared to those shot at the landfill.
(0.04 MB DOC)
