# Supplementary material for: The Young, the Weak and the Sick: Evidence of Natural Selection by Predation
Source: PLoS One. 2010 Mar 19;5(3):e9774. doi: 10.1371/journal.pone.0009774 (PMC2841644; doi:10.1371/journal.pone.0009774)
Supplement: Table S1 — Model selection of individual features of gulls predated by birds of prey compared to those shot at the landfill. (0.04 MB DOC) [file pone.0009774.s001.doc]

Table S1. Model selection of individual features of gulls predated by birds of prey compared to those shot at the landfill. ΔAIC = difference AIC value versus the best model; *w*_i_ = weight for model *i*; MuscCond = muscle condition.

| **Model** | **AIC** | **ΔAIC** | ***w*_i_** |
| --- | --- | --- | --- |
| MuscCond + Age + Sickness | 417.38 | 0 | 0.39 |
| MuscCond + Age + Sickness + Sex | 417.69 | 0.31 | 0.34 |
| MuscCond + Age + Sickness + Parasites | 419.37 | 1.99 | 0.14 |
| MuscCond + Age + Sickness + Sex + Parasites | 419.63 | 2.25 | 0.13 |
| MuscCond + Age+ Sex | 434.33 | 16.95 | 0 |
| MuscCond + Age | 434.86 | 17.48 | 0 |
| MuscCond + Sickness + Sex | 445.88 | 28.5 | 0 |
| MuscCond + Sickness | 447.98 | 30.6 | 0 |
| MuscCond | 473.59 | 56.21 | 0 |
| Age+ Sickness | 542.49 | 125.11 | 0 |
| Age+ Sickness + Sex | 542.5 | 125.12 | 0 |
| Age | 569.13 | 151.75 | 0 |
| Sickness | 584.76 | 167.38 | 0 |
| Sex | 614.84 | 197.46 | 0 |
| Null | 618.39 | 201.01 | 0 |
